# Supplementary material for: Restricted cell cycle is essential for clonal evolution and therapeutic resistance of pre-leukemic stem cells
Source: Nat Commun. 2018 Aug 30;9:3535. doi: 10.1038/s41467-018-06021-7 (PMC6117297; doi:10.1038/s41467-018-06021-7)
Supplement: Supplementary file 1 — Supplementary Information [file 41467_2018_6021_MOESM1_ESM.pdf]

Restricted cell cycle is essential for clonal evolution and therapeutic resistance of pre-leukemic stem cells

Tremblay, *et al*.

SUPPLEMENTARY FIGURES……………………………………………………..…………………………2

Supplementary Figure 1. GFP-labelled populations of thymocytes in H2B-GFP mice……………………2

Supplementary Figure 2. Exclusive features of cell-cycle restricted pre-LSCs……………………………3

Supplementary Figure 3. Gene expression profiling of GFP-labelled DN3 thymocytes…………………..4

Supplementary Figure 4. Genetic abnormalities of GFP-labelled DN3 thymocytes……………………….5

Supplementary Figure 5. Effect of loss of p21 in pre-LSCs……………………………..……………………6

Supplementary Figure 6. Effect of absence of p21 in DN3 thymocytes………………………………...……8

SUPPLEMENTARY METHODS………………………………………………………………………….9

SUPPLEMENTARY REFERENCES……………………………………………………………………10

Supplementary Figures

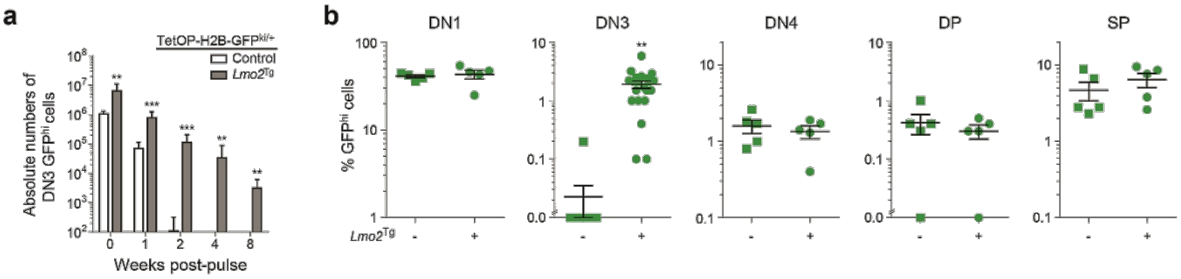

Supplementary Figure 1. GFP-labelled populations of thymocytes in H2B-GFP mice. a, Absolute numbers of GFP^{hi} DN3 thymocytes from *H2B-GFP;Lmo2*^{Tg} mice and littermate controls after labelling, followed by 0, 1, 2, 4 or 8 weeks of chase without Doxycycline, as indicated. **b,** Proportion (%) of GFP^{hi} cells in different thymocyte populations in H2B-GFP mice 2 weeks after Doxycycline pulse. Mean ± SD, Student's *t*-test; **p<0.01 and ***p<0.001, as compared to *H2B-GFP* controls.

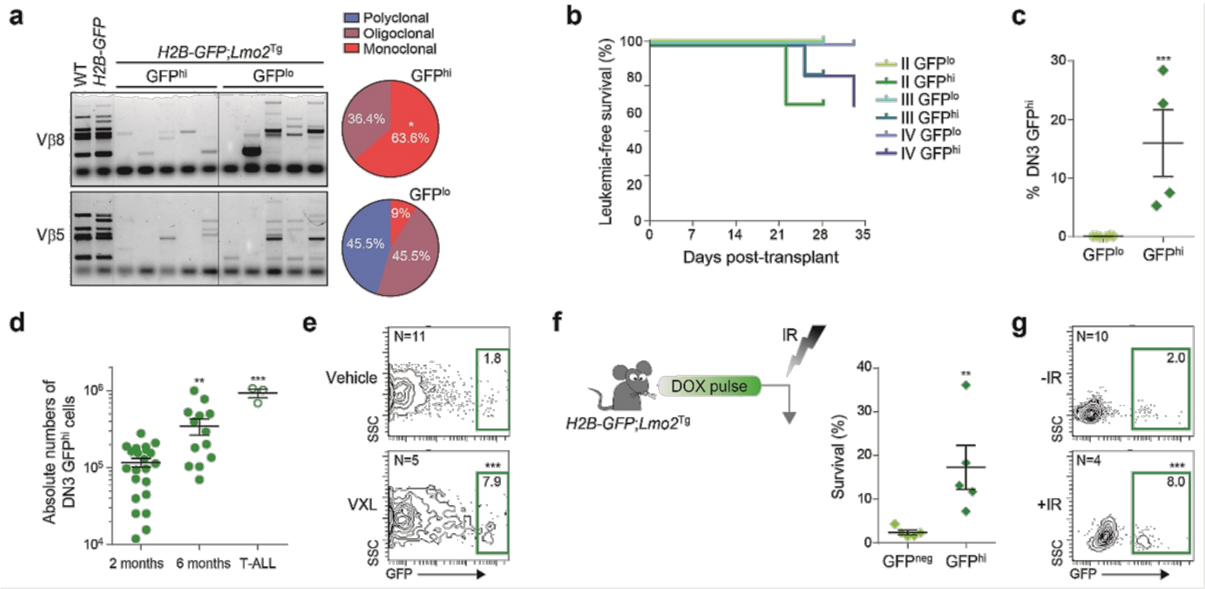

Supplementary Figure 2. Exclusive features of cell-cycle restricted pre-LSCs. a, *Tcr*β gene rearrangement analysis of in matching purified GFP^{lo} and GFP^{hi} DN3 thymocytes from 2-month old *H2B-GFP;Lmo2*^{Tg} mice (panel) and proportion of mono-, oligo- and poly-clonal *Tcr*β gene rearrangements (pie charts) in these purified thymocytes (n = 7). Two-way ANOVA test with a Two-stage linear step-up procedure of Benjamin, Krieger and Yekutieli. **b,** Kaplan-Meier curves of the time to leukemia for recipients serially transplanted with purified GFP^{lo} and GFP^{hi} DN3 thymocytes from *H2B-GFP;Lmo2*^{Tg} mice. All malignant thymic tumours were diagnosed at necropsy. **c,** Proportion of donor-derived GFP^{hi} DN3 cells in the thymus of primary recipients injected with purified GFP^{lo} and GFP^{hi} DN3 thymocytes, 2 weeks post-pulse. Mean ± SD, Student's *t*-test. **d,** Absolute numbers of GFP^{hi} DN3 cells in the thymus of 2-month, 6-month old and leukemic *H2B-GFP;Lmo2*^{Tg} mice following 2 weeks of chase after labelling with doxycycline. Mean ± SD, Student's *t*-test; **e** and **g**, Proportion (%) of GFP^{hi} DN3 thymocytes from *H2B-GFP;Lmo2*^{Tg} mice 24 hours after treatment with VXL induction-like therapy (**e**) or sublethal irradiation (IR, **g**), following 2 weeks of chase after labelling with doxycycline. Mean, Student's *t*-test. **f,** Proportion (%) of radioresistant DN3 thymocytes from each GFP-labelled subsets found in *H2B-GFP;Lmo2*^{Tg} mice 2 weeks after Doxycycline pulse. Mean ± SD, Student's *t*-test **$p<0.01$, **$p<0.01$, ***$p<0.001$.

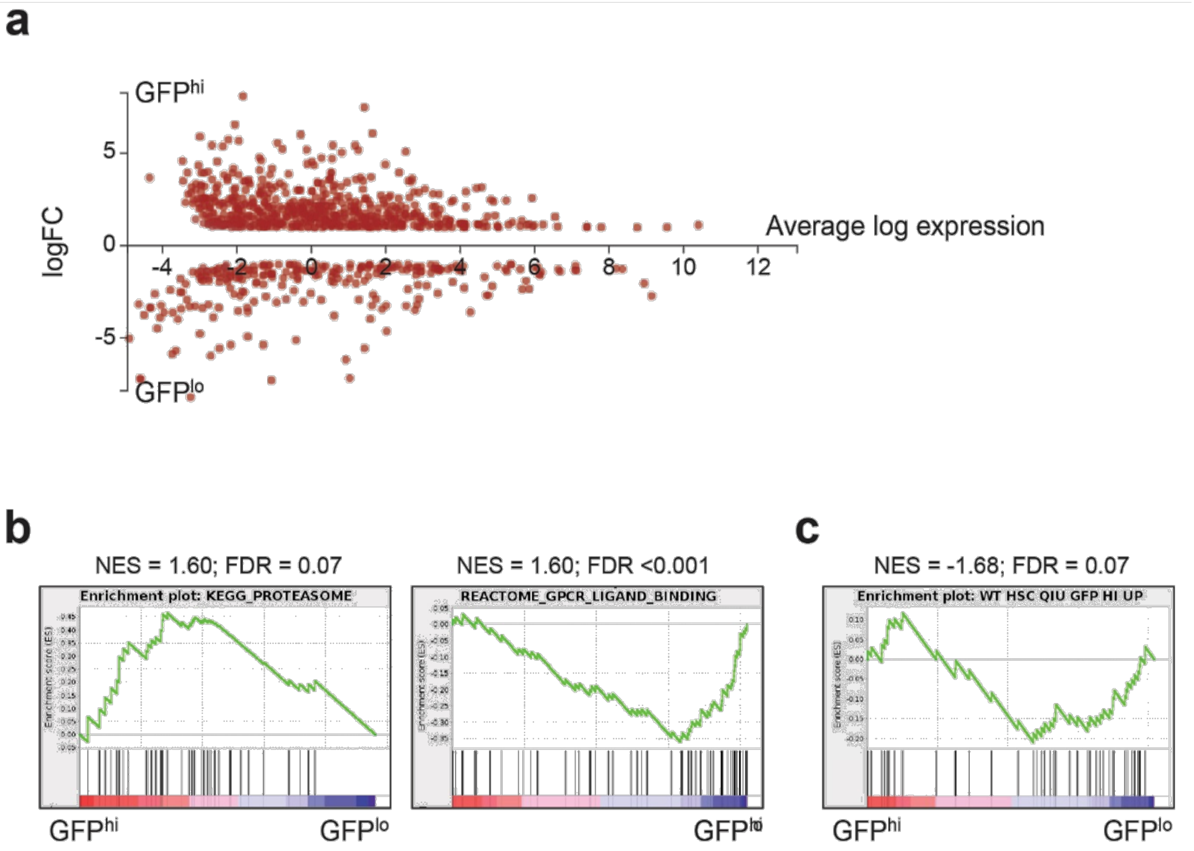

Supplementary Figure 3. Gene expression profiling of GFP-labelled DN3 thymocytes. a, MA plot of differentially expressed genes (LogFC>2; FDR<0.01) in purified GFP^{lo} and GFP^{hi} DN3 thymocytes from 2-month old *H2B-GFP;Lmo2*^{Tg} mice. Average expression for each sample analysed was used as reference. Gene set enrichment analysis (GSEA) of **b,** proteasome activation genes and antigen processing pathway (GPCR_ligand_binding), as well as **c,** cell cycle genes upregulated in quiescent GFP^{hi} HSCs from[1]. FDR: false discovery rate; NES: normalized enrichment score in GFP^{hi}, as compared to GFP^{neg} population.

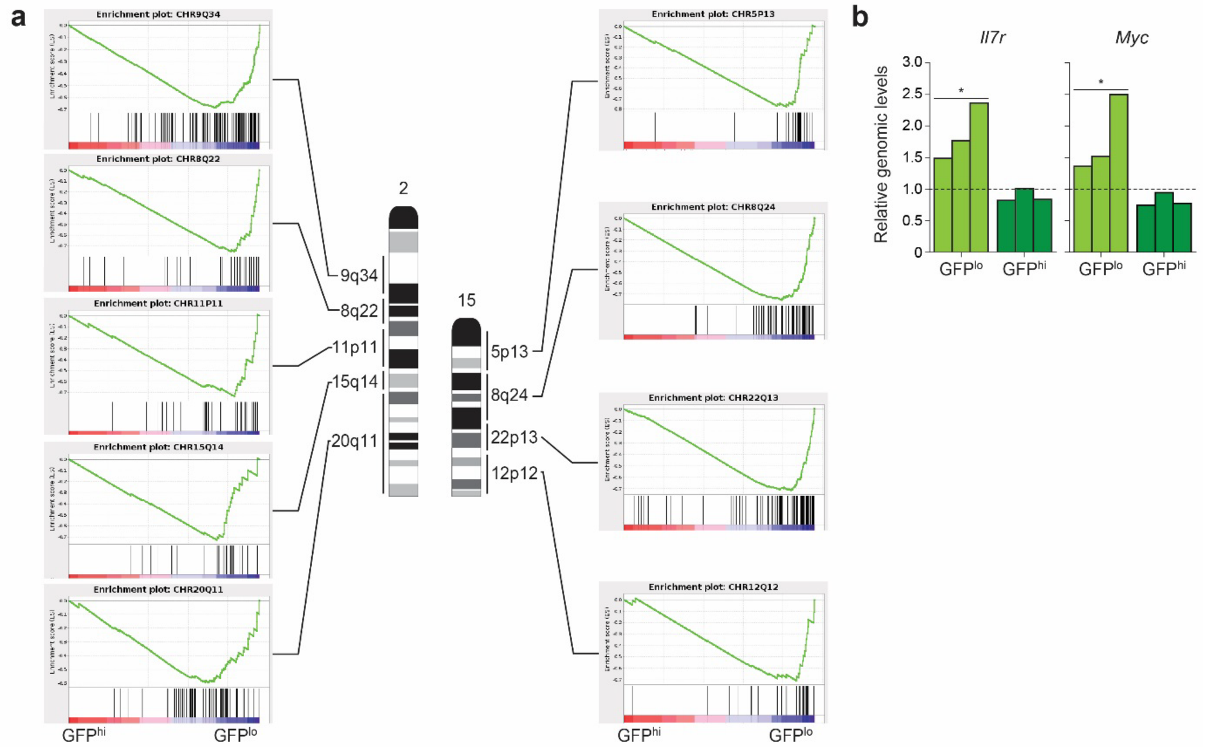

Supplementary Figure 4. Genetic abnormalities of GFP-labelled DN3 thymocytes. a, Gene set enrichment analysis (GSEA) of genes found on human chromosomes that clustered on murine chromosomes 2 and 15 (represented in the centre). FDR: false discovery rate; NES: normalized enrichment score in GFP^{hi}, as compared to GFP^{neg} population. **b**, Comparative genomic quantification by qRT-PCR analysis for the *Il7r* and *Myc* loci, which are present on murine chromosome 15. Student's *t*-test *p<0.05, as compared to the expression in wild-type thymocytes.

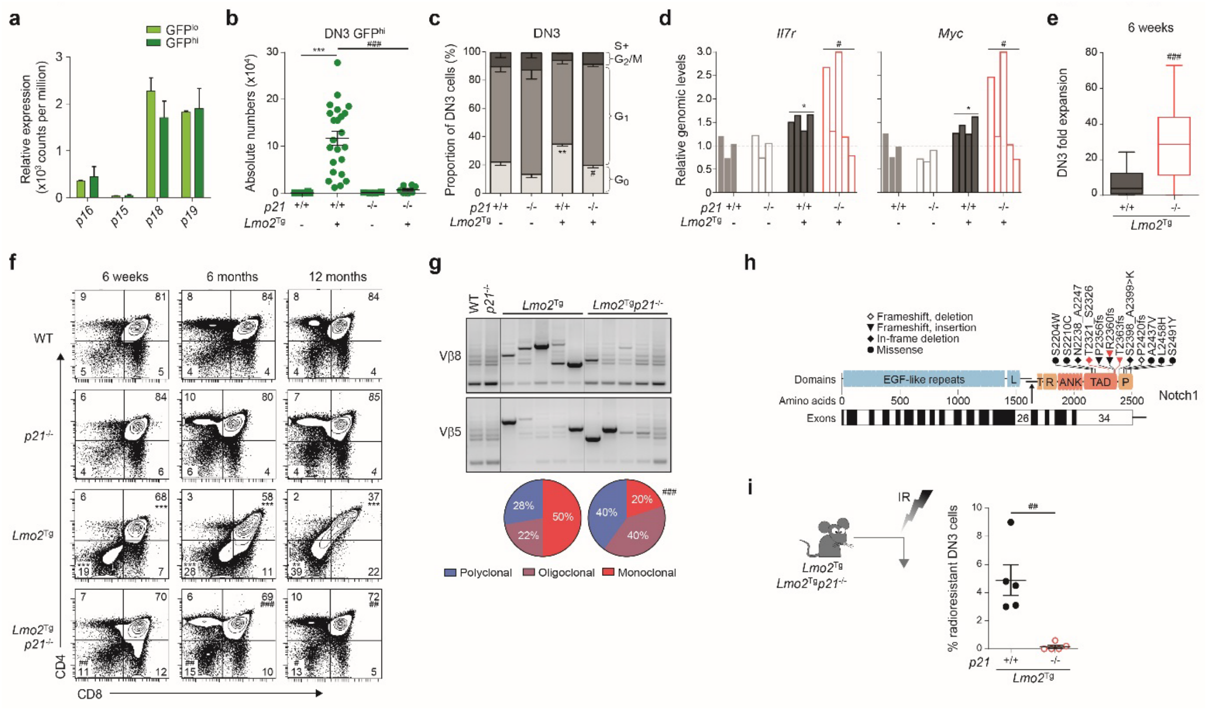

Supplementary Figure 5. Effect of loss of p21 in pre-LSCs. a, Gene expression by qRT-PCR analysis of Cdkn2 cell cycle regulators in GFP^{lo} and GFP^{hi} DN3 thymocytes from *H2B-GFP;Lmo2*^{Tg} mice 2 weeks after Doxycycline pulse. Mean ± SD, Student's *t*-test. **b**, Absolute numbers of GFP^{hi} DN3 thymocytes in 6 week-old wild-type (WT), *p21*$^{-/-}$, *Lmo2*^{Tg} and *Lmo2*^{Tg}*;p21*$^{-/-}$ mice on a H2B-GFP background after 2 weeks of chase. Mean ± SD, Two-way ANOVA test with Turkey's correction. **c**, Cell cycle analysis of DN3 thymocytes in 6 week-old wild-type (WT), *p21*$^{-/-}$, *Lmo2*^{Tg} and *Lmo2*^{Tg}*;p21*$^{-/-}$ mice. Mean ± SEM, Two-way ANOVA test with Turkey's correction. **d**, Comparative genomic quantification by qRT-PCR analysis for the *Il7r* and *Myc* loci, which are present on murine chromosome 15 in thymocytes from 6 week-old wild-type (WT), *p21*$^{-/-}$, *Lmo2*^{Tg} and *Lmo2*^{Tg}*;p21*$^{-/-}$ mice. Student's *t*-test. **e**, Fold expansion of donor-derived DN3 thymocytes from 6-week old *Lmo2*^{Tg} and *Lmo2*^{Tg}*;p21*$^{-/-}$ mice in transplanted recipients. Student's *t*-test. **f**, Representative flow cytometry analysis of the thymus of 6-week, 6-month and 12-month old mice. Mean, Student's *t*-test. **g,** *Tcrβ* gene rearrangement analysis of thymocytes from 6-month old mice (panel) and proportion of mono-, oligo- and poly-clonal *Tcrβ* gene rearrangements (pie charts) in *Lmo2*^{Tg} (n = 8) and *Lmo2*^{Tg}*;p21*$^{-/-}$ (n = 6) thymocytes. Two-way ANOVA test with a Two-stage linear step-up procedure of Benjamin, Krieger and Yekutiel. **h,** Diagram showing the position of mutations found in the *Notch1* gene sequence in DN3 thymocytes from 6-month old *Lmo2*^{Tg} (black symbols, n=9) and *Lmo2*^{Tg}*;p21*$^{-/-}$ mice (red symbols, n=5). Wild-type (WT, n=3), *p21*$^{-/-}$ (n=3) were used as reference sequence. The predicted sequence change is shown, with their position

in the corresponding exons from the *Notch1* locus. L: Lin/NOTCH repeats, T: transmembrane domain, RAM: RAM domain, ANK: Ankyrin repeat domain, TAD: transactivation domain, PEST: PEST domain, and the arrow indicated the site of cleavage releasing the Notch1 intracellular domain following activation. **i,** Proportion (%) of radioresistant DN3 thymocytes, 24 hours after total body irradiation. Mean ± SD, Student's *t*-test. *p<0.05, ***p<0.001, as compared to WT; $^{\#}$p<0.05, $^{\#\#}$p<0.01, $^{\#\#\#}$p<0.001, as compared to *Lmo2*^{Tg} mice.

a

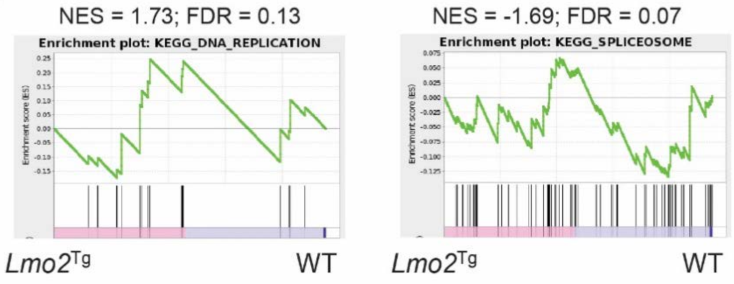

b

	Numbers		*P* values		
Groups	SCD	ACD	Reference used		
			WT	*$p21^{-/-}$*	*$Lmo2^{Tg}$*
WT	69	36			
$p21^{-/-}$	103	39	0.3		
$Lmo2^{Tg}$	44	53	0.004	<0.0001	
$Lmo2^{Tg}p21^{-/-}$	64	43	0.3	0.02	0.05

Supplementary Figure 6. Effect of absence of p21 in DN3 thymocytes. a, Gene set enrichment analysis (GSEA) of DNA replication and splicing genes in *$Lmo2^{Tg}$* DN3 thymocytes as compared to wild-type (WT) controls. FDR: false discovery rate; NES: normalized enrichment score in *$Lmo2^{Tg}$*, as compared to WT. **b,** Contingency table and Chi-Square test described for each genotype, as indicated. Number of cells for each genotype (Groups) and cell division patterns are indicated; SCD: symmetric cell division; ACD: asymmetric cell division. Significant differences are in red.

Supplementary Methods

***Tcrβ* gene rearrangement analysis.** Genomic DNA was obtained from thymocytes using Blood & Cell Culture DNA Mini kit (13323, Qiagen, Chadstone VIC, Australia), according to the manufacturer protocol. *Tcrβ* gene rearrangements were determined by PCR amplification, as previously described[4], using the primers listed in Table 1.

Genomic quantification of the *Il7r* and *Myc* loci. Genomic DNA was obtained from thymocytes using Blood & Cell Culture DNA Mini kit (13323, Qiagen, Chadstone VIC, Australia), according to the manufacturer protocol. Genomic PCR for the *Il7r* and *Myc* loci were performed using the primers listed in Table 1.

Supplementary References

1. Qiu J, Papatsenko D, Niu X, Schaniel C, Moore K. Divisional history and hematopoietic stem cell function during homeostasis. *Stem cell reports* **2**, 473-490 (2014).

2. Henriques CM, Rino J, Nibbs RJ, Graham GJ, Barata JT. IL-7 induces rapid clathrin-mediated internalization and JAK3-dependent degradation of IL-7Ralpha in T cells. *Blood* **115**, 3269-3277 (2010).

3. Pham K*, et al.* Asymmetric cell division during T cell development controls downstream fate. *The Journal of cell biology* **210**, 933-950 (2015).

4. Tremblay M*, et al.* Modeling T-cell acute lymphoblastic leukemia induced by the SCL and LMO1 oncogenes. *Genes & development* **24**, 1093-1105 (2010).
