# Supplementary material for: Restricted cell cycle is essential for clonal evolution and therapeutic resistance of pre-leukemic stem cells
Source: Nat Commun. 2018 Aug 30;9:3535. doi: 10.1038/s41467-018-06021-7 (PMC6117297; doi:10.1038/s41467-018-06021-7)
Supplement: Supplementary file 3 — Description of Additional Supplementary Files [file 41467_2018_6021_MOESM3_ESM.pdf]

Description of Additional Supplementary Files

File Name: Supplementary Data 1
Description: Differential gene expression in GFPhi vs GFPlo DN3 cells. **Gene list** Genes differentially expressed more than 2-fold (LogFC>1 or <-1) using a False Discovery Rate (FDR) of 0.01 in GFPhi DN3 cells compared to GFPlo cells from *H2B-GFP;Lmo2Tg* mice. **GO UP** Gene ontology (GO) analysis of genes increased in GFPhi DN3 cells. **GO DOWN** GO analysis of genes decreased in GFPhi DN3 cells. **GSEA UP** Gene Set Enrichment Analysis (GSEA) of genes increased in GFPhi DN3 cells using the REACTOMEv2.0 genesets. **GSEA DOWN** GSEA of genes decreased in GFPhi DN3 cells using the REACTOMEv2.0 genesets. **Genesets HSC QIU GFPhi v lo** Genes increased and decreased in GFP-retaining mouse bone marrow Lin-Sca+cKit+ cells (GFP4 population) compared to GFP0-1 population 29 using GEO2R analysis program. **GSEA QIU DOWN** GSEA of genes decreased GFPhi DN3 cells using the Qiu downregulated genes.

File Name: Supplementary Data 2
Description: Variants expressed in RNA-seq. **Sheet Variants RNA-seq GFPhi v lo** Variants differentially expressed in GFPhi DN3 cells, as compared to GFPlo cells from *H2B-GFP;Lmo2Tg* mice. L = GFPlo sample; H = GFPhi sample; numbers listed below L3, L2, L1, H3 and H1 are the counts obtained for each gene, followed by the counts for the variant in each sample. Example: 10,2 = 10 counts for the gene, 2 counts for the variant (20% frequency). Data for variants only present in GFPhi cells are highlighted in yellow. **Sheet Variants RNA-seq Lmo2 v Lmo2p21** Variants differentially expressed in DN3 cells from 6-week old wild-type (WT), *p21-deficient (p21-/-)*, *Lmo2Tg* and *Lmo2Tgp21-/-* mice. Data for Notch1 variants are highlighted in yellow. Chrom: Chromosome location of the gene variants; GeneName: name of the gene variant; Pos: position of the mutation; Ref: reference sequence of the gene; Alt: mutated sequence.

File Name: Supplementary Data 3
Description: Differential gene expression in DN3 cells. **Sheet LogFC>1or<-1 p21 v wt** Genes differentially expressed more than 2-fold (LogFC>1 or <-1) in p21-deficient DN3 cells, as compared to wild-type (wt) controls. **LogFC>1or<-1 Lp21 v L** Genes differentially expressed more than 2-fold (LogFC>1 or <-1) using a False Discovery Rate (FDR) of 0.01 in DN3 cells from *Lmo2Tg;p21-/-* mice, as compared *to Lmo2Tg* mice. GO DOWNregulated Lp21 v L Gene ontology (GO) analysis of downregulated genes in *p21-deficient Lmo2Tg* DN3 cells, as compared to DN3 cells from *Lmo2Tg* mice.
